# Novel Exfoliation of High-Quality 2H-MoS_2_ Nanoflakes for Solution-Processed Photodetector

**DOI:** 10.3390/nano10061045

**Published:** 2020-05-29

**Authors:** Seulgi Kim, Woojin Park, Dohoon Kim, Jiyeon Kang, Jaesoung Lee, Hye Yeon Jang, Sung Ho Song, Byungjin Cho, Dongju Lee

**Affiliations:** 1 Department of Advanced Materials Engineering, Chungbuk National University, Chungdae-ro 1, Seowon-Gu, Cheongju 28644, Korea; kimsg@chungbuk.ac.kr (S.K.); wjpark80@gmail.com (W.P.); rlaehgns235@gmail.com (D.K.); wldusk0609@chungbuk.ac.kr (J.K.); jaesounglee@cbnu.ac.kr (J.L.); hyjang0581@gmail.com (H.Y.J.); 2 Division of Advanced Materials Engineering, Kongju National University, Kongju, Chungnam 330-717, Korea

**Keywords:** molybdenum disulfide, nanoflakes, hydrazine, ball milling, photodiode

## Abstract

Highly dispersive molybdenum disulfide nanoflakes (MoS_2_ NFs), without any phase transition during the exfoliation process, are desirable for full utilization of their semiconductor properties in practical applications. Here, we demonstrate an innovate approach for fabricating MoS_2_ NFs by using hydrazine-assisted ball milling via the synergetic effect of chemical intercalation and mechanical exfoliation. The NFs obtained have a lateral size of 600–800 nm, a thickness less than 3 nm, and high crystallinity in the 2H semiconducting phase. They form a stable dispersion in various solvents, which will be helpful for many applications, due to the oxygen functional group. To investigate production of a two-dimensional (2D) photodetector, 2D semiconducting MoS_2_, MoS_2_–p-Si vertical devices were fabricated, and their optical properties were characterized. The photodiode exhibited consistent responses with excellent photo-switching characteristics with wavelengths of 850, 530, and 400 nm.

## 1. Introduction

Transition metal dichalcogenides (TMDs) are layered materials composed of chemicals with the formula MX_2_ (M = Mo, W, Hf, Ti, Nb, or V; X = S, Se, or Te) [1,2]. Due to the different combinations of transition metal and chalcogen atoms, more than 40 types of TMDs with a layered structure have been investigated [3]. As the most representative TMDs, two-dimensional (2D) molybdenum disulfide (MoS_2_) has gained a lot of research interest because of its unique optical and electrical characteristics, originating from the various crystal structures and different electronic configurations. There are three typical MoS_2_ crystal structures: 1T (D_3d_ group, AA stack), 2H (D_3H_ group, ABAB stack) and 3R (C^5^_3v_ group, ABCABC stack) [4]. Of these, 1T-MoS_2_ is suitable for catalyst applications due to its metallic properties, and 2H-MoS_2_ has a bandgap suitable for electronic and optical applications requiring semiconductor characteristics, including field effect transistors [5,6], optical detectors [7,8], gas sensors [9,10,11], and others [12,13,14]. Remarkably, to take full advantage of these outstanding semiconductor properties of MoS_2_, its 2H structure should be maintained, without phase conversion, throughout the exfoliation process.

To date, several methods, have been introduced to obtain few-layered TMDs, including mechanical exfoliation [15], liquid phase exfoliation [16,17,18,19], alkali ion intercalation [20,21], chemical vapor deposition (CVD) [22,23], and ball milling [24]. Each technique has pros and cons. Mechanical exfoliation can lead to high-quality MoS_2_ flakes, but cannot easily be scaled up [25]. Sonication-assisted liquid phase exfoliation is a simple process, but the obtained MoS_2_ flakes usually cause a dispersion issue in solvents other than those used during the process [26]. Alkali ion intercalation is a process whereby alkali ions (Li^+^, Na^+^, and K^+^) penetrate into the van der Waals gap of the bulk MoS_2_ layers and then separate these layers. This method makes it easier to achieve a relatively thin layer, but often results in a crystal structure conversion from the 2H semiconducting phase to the 1T metallic phase [20]. CVD can synthesize wafer-sized monolayer MoS_2_, but it requires transfer processes and a high temperature [22]. Ball milling is suitable for mass production, but it destroys or disorders the crystal structure, generating a very large number of defects [24]. Therefore, developing a large-scale and simple way to synthesize high-quality and highly soluble MoS_2_ flakes remains a significant challenge.

Herein, we present a method for easily fabricating MoS_2_ nanoflakes (NFs) by hydrazine-assisted ball milling via the synergistic effect of chemical intercalation and mechanical exfoliation. The key idea of this exfoliation method is that hydrazine (N_2_H_4_) and 1-methyl-2pyrrolidone (NMP) molecules, which play the roles of intercalant and co-intercalant, promote the exfoliation process by expanding the interlayer when the shear force of the ball is applied to MoS_2_. In addition, the liquid intercalant can improve the quality of MoS_2_ NFs by reducing the impact energy that can occur during the ball milling process. This hydrazine-assisted ball milling method maintains the 2H semiconducting structure, even after the exfoliation process, and produces high-quality MoS_2_ NFs. The NFs exhibited excellent dispersibility in various solvents. To the best of our knowledge, the work described here represents the first demonstration of highly crystalline MoS_2_ NFs, with a large lateral size and a thickness of a few layers, using ball milling exfoliation methods. In contrast, previously reported NFs produced via ball milling had high defect densities, which worsens their opto-electronic properties, and were of small sizes, like quantum dots. We also confirmed the applicability of our NFs for use in optoelectronics by evaluating an MoS_2_–Si stacked photodiode.

## 2. Materials and Methods

### 2.1. Materials

MoS_2_ powder was purchased from Sigma-Aldrich (Merck, Darmstadt, Germany). NMP, N,N-dimethylformamide, hydrochloric acid (HCl), dimethyl sulfoxide, and ethanol were purchased from Samchun Chemical, Inc (Seoul, Republic of Korea). Isopropyl alcohol and hydrazine monohydrate were purchased from JUNSEI (Tokyo, Japan). All chemicals were used without further purification.

### 2.2. Exfoliation of MoS_2_

In total, 2 g of as-received MoS_2_ (lateral size: 10 µm) powder, 20 mL of mixed solution (NMP:N_2_H_4_ = 19:1) and 100 g of steel balls (8 mm diameter) were placed into a steel jar for a horizontal planetary mill (Pulverisette 5, Fritsch, Idar-Oberstein, Germany). The exfoliation process was conducted at a rotational speed of 200 rpm for 48 h. After milling, the product was collected and residual contaminants, such as Fe ions, were removed with HCl solution (100 mL) and rinsed with DI several times, until the pH was seven. The product was dispersed in NMP (1 L) by ultrasonication for 90 min, and then centrifuged for 30 min at 2000 rpm to remove unexfoliated and thick flakes.

### 2.3. Fabrication of MoS_2_ Photodiode Device

The MoS_2_ NFs layer was prepared using the Langmuir–Blodgett method on strongly p-type doped silicon wafer, covered with 100 nm-thick SiO_2_. NFs dispersed in NMP were slowly spread on the surface of some water, and then, this NF layer was transferred onto the p-Si substrate. Using sputtering with a shadow mask with a width of 500 µm and a gap of 50 µm, Ti and Au electrodes were deposited on the NF layer to 5 and 70 nm, respectively. The photo-electrode contact pad size is 200 × 500 μm^2^

### 2.4. Characterization of MoS_2_ NFs

The microstructure of the MoS_2_ NFs was examined using field-emission SEM (FE-SEM; Crossbeam 540, ZEISS, Ulm, Germany) and TEM (JEM-ARM200F, JEOL, Tokyo, Japan). TEM samples were prepared by drying a droplet of the MoS_2_ NF suspension on a Lacey carbon grid. For AFM (SPM-9700 from Shimadzu, Kyoto, Japan), NFs were deposited on a Si substrate by spin-coating. XPS measurements were carried out on a Quantera-Ⅱ system from ULVAC-PHI (Chigasaki, Kanagawa, Japan). TGA (TGA92-18, Setaram, Caluire, France) was used to determine the weight of functional groups under an N_2_ atmosphere at a heating rate of 10 °C/min. A UV–visible analysis of dispersibility was conducted (Cary-5000, Agilent, Santa Clara, CA, USA). Raman spectra were measured using a micro-Raman spectrometer (RAMANtouch, Nanophoton, Bundang-gu, Republic of Korea). XRD patterns were obtained (JP/SmartLab, Rigaku, Tokyo, Japan) at a power of 9 kW.

### 2.5. Characterization of MoS_2_ Photodiode Device

All measurements were carried out in ambient conditions at room temperature. The I–V characteristics of MoS_2_ devices were analyzed (2636B, Keithley, Cleveland, OH, USA) under dark conditions. Photo measurements (4200-SCS, Keithley, Cleveland, OH, USA) were conducted with laser sources with wavelengths of 400, 530, and 850 nm providing illumination to observe photosensitivity. The morphology of the device was analyzed with via AFM (Dimension ICON, Bruker, Bremen, Germany) and the same FE-SEM as above.

## 3. Results

### 3.1. Fabrication and Mechanism Analysis of MoS_2_ Nanoflakes

**Figure 1:**
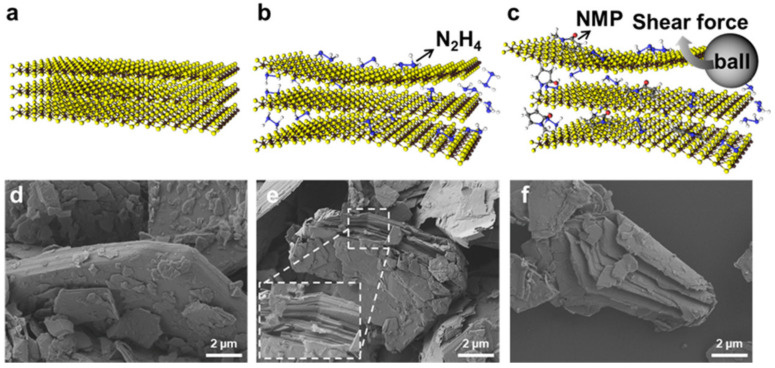
Schematic illustration and corresponding scanning electron microscopy images of the exfoliation mechanism of MoS_2_. (**a**,**d**) As-received MoS_2_. (**b**,**e**) Hydrazine is intercalated into the layer, to form an expanded MoS_2_ structure. (**c**,**f**) Exfoliation due to the shear force of the ball as additional 1-methyl-2pyrrolidone (NMP) molecules are intercalated.

Figure 1 presents a schematic illustration and corresponding scanning electron microscopy (SEM) images representing the exfoliation process of as-received MoS_2_ by hydrazine-assisted ball milling. The N_2_H_4_ molecules are intercalated into the as-received MoS_2_, and are then partially oxidized to N_2_H_5_^+^, which is thermally unstable and decomposes into gases including N_2_, NH_3_, and H_2_ during the process. Decomposition and gasification of these intercalated molecules expand the interlayer spacing of the MoS_2_ (Figure 1b,e) [27]. The interlayer is further expanded by intercalated NMP molecules, which have a larger molecular size than that N_2_H_4_. Next, NFs are exfoliated from the expanded MoS_2_ particles from the shear force by the rotation of the ball (Figure 1c,f).

Liquid control agents (NMP solutions in this case) are essential for milling and serve as lubricants, reducing impact energy, and thus, limiting damage to the MoS_2_ structure, and preventing restacking during milling [28]. Milling was optimized by controlling the rotation speed (rpm) and milling time. As the rpm increased, the number of MoS_2_ layers decreased, but the lateral size decreased (Figure S1). As the milling time increased, the number of layers also decreased, although small lateral size and poor crystallinity were obtained after 48 h (Figure S2).

### 3.2. Morphology Analysis of MoS_2_ Nanoflakes

**Figure 2:**
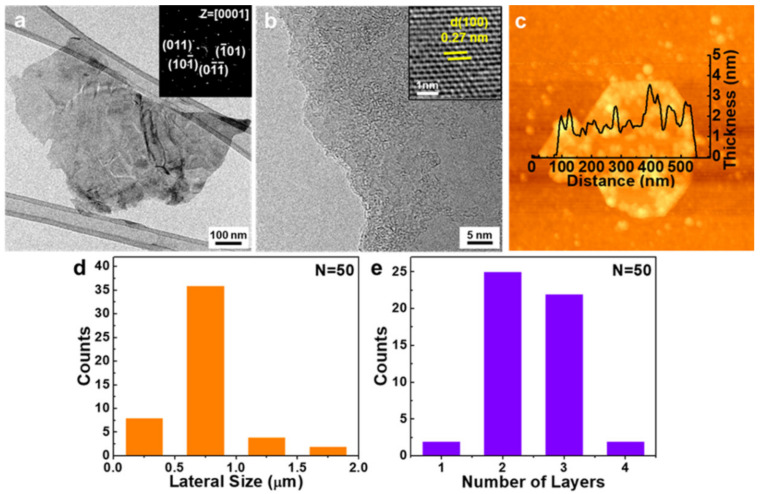
(**a**) Low-magnification transmission electron microscopy (TEM) images of MoS_2_ nanoflakes (NFs) and corresponding selected area electron diffraction (SAED) patterns. (**b**) High-resolution TEM (HRTEM) images of MoS_2_ NF edges. (**c**) Atomic force microscopy (AFM) topography image of MoS_2_ NFs. Histogram data representing the distributions of the (**d**) lateral size and (**e**) number of layers for 50 flakes.

The morphology of the MoS_2_ NFs was characterized by transmission electron microscopy (TEM) and atomic force microscopy (AFM). Figure 2a presents a low-magnification TEM image verifying the presence of thin NFs. The inset of Figure 2a shows a selected area electron diffraction (SAED) pattern. The pattern exhibits the typical six-fold symmetry of 2H-MoS_2_, indicating that the hexagonal structure of the NFs received little damage during the hydrazine-assisted ball milling process. As shown in Figure 2b, the number of layers on the edges of the NFs was estimated to be approximately two to three. The inset of Figure 2b presents high-resolution TEM (HRTEM) images. The lattice distance was measured to be ~0.27 nm, which corresponds to the (100) lattice plane of 2H-MoS_2_. The morphologies and thicknesses of NFs were also characterized by scanning them via AFM, as shown in Figure 2c. The average topographic height was ~2.2 nm, which corresponds to the typical height of a three-layer MoS_2_. The histograms shown in Figure 2d,e present the distribution of lateral size and number of layers of the NFs. The lateral size was approximately 600–800 nm, and the NFs mostly comprised thin flakes with two to three layers. The structure of NFs was also assessed using Raman spectroscopy (Figure S3). The frequency difference, which is the interval between the two peaks, is ~23 cm^−1^, indicating that they have three layers on average.

### 3.3. Chemical Analysis of MoS_2_ Nanoflakes

**Figure 3:**
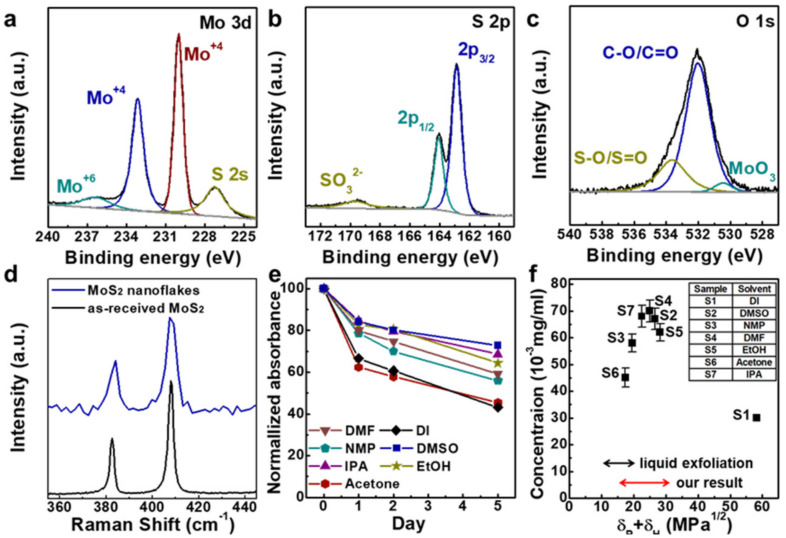
XPS narrow scan of (**a**) Mo 3d, (**b**) S 2p, and (**c**) O 1s. (**d**) Raman spectra of as-received MoS_2_ and MoS_2_ NFs. (**e**) Dispersibility of MoS_2_ NFs over time in each solvent. (**f**) Dispersibility and Hansen solubility parameters of MoS_2_ NFs for each tested solvents.

The chemical state of NFs was confirmed using X-ray photoelectron spectroscopy (XPS; Figure 3 and Figure S5). In the Mo 3d narrow scan, two distinct peaks at 232.9 and 229.7 eV correspond to Mo^4+^ 3d_3/2_ and Mo^4+^ 3d_5/2_ of the hexagonal MoS_2_ phase, respectively (Figure 3a). In previous reports regarding liquid phase exfoliation and alkali ion intercalation, the Mo^+6^ peak at 235.4 eV from MoO_3_ increased significantly; the corresponding peak is hardly observed in the XPS result of MoS_2_ NFs prepared by our hydrazine-assisted ball milling, which suggests that our process prevented oxidation of the NFs [29,30]. The data for the S 2p core level shown in Figure 3b were deconvolved into two peak components: double peaks at 163.8 and 162.6 eV corresponded to S 2p_1/2_ and S 2p_3/2_, respectively [31].

An S vacancy is easily formed at MoS_2_ edge sites during the exfoliation process due to the intrinsic characteristics of the material’s crystal structure. Partial MoO_3_ is typically formed by attachment of O atoms to the S vacancy sites, which distort the MoS_2_ crystal structure. Figure 3c presents the O narrow scan spectra, with the peak for S–O bonding at 533.5 eV and the peak for MoO_3_ at 530.7 eV. Compared with the as-received MoS_2_ XPS spectra, these peaks slightly increased after exfoliation, as shown in Figure S4. The oxygen content was identified as constituting 3.96% of what was nominally sulfur and 0.89% of molybdenum in the MoS_2_ NFs, compared to 1.61% of sulfur and 0.05% of molybdenum in the as-received MoS_2_. Oxygen atoms not only substitute in the S vacancies forming Mo–O bonds, but also attach to S, forming S–O bonds. Moreover, additional peaks indicating the presence of tetragonal 1T-MoS_2_ were not found at their expected 231.8 and 228.6 eV in the Mo narrow scan. Therefore, it can be concluded that the oxygen on the MoS_2_ NFs obtained by our process acts as a functional group without distorting the original lattice structure. Moreover, the XPS spectra present no evidence of any other elements in MoS_2_ NFs (Figure S5). Thermogravimetric analysis (TGA) was also performed, to determine the mass fraction of the oxygen functional groups in the NFs in an N_2_ atmosphere. The results revealed that ~5 wt% of these existed, comparable to the XPS result (Figure S6).

The presence of oxygen functional groups on the NFs was also demonstrated by Raman spectroscopy (Figure 3d). Peaks corresponding to MoS_x_O_y_ were observed at 380.15 and 403.37 cm^−1^, which was not the case for the as-received MoS_2_ [32,33]. Additionally, X-ray diffractometer (XRD) analysis gave further information on the crystallinity of the NFs (Figure S7).

Simple solutions are essential for many applications, so it is important to fabricate MoS_2_ NFs with high dispersibility. Their maximum dispersibility depends both on the solvent and the degree of functionalization imparted during exfoliation [34]. The dispersion properties of our MoS_2_ NFs at equal concentrations were measured in seven solvents for a period of more than one week (Figure S8); their Hansen solubility parameters were also considered. The concentration of each dispersion was estimated based on the mass of NFs in that dispersion. As shown in Figure S8, absorbance increases linearly with increasing MoS_2_ concentration, indicating that the NFs exhibit Lambert–Beer behavior in various solvents. In the Tauc plot in Figure S8d, the optical band gap is measured to 1.6 eV, corresponding to several layers of MoS_2_ NF. Figure 3e presents the degree of dispersion over time in various solvents at a nominal concentration of 0.1 mg/mL; the dispersions were allowed to settle for one week. The concentration of NFs remained above 60% of the initial value for each solvent except deionized water (DI) and acetone, but it remained above 40% for both of these. Figure 3f and Figure S6 present the Hansen solubility parameters of MoS_2_ obtained by liquid exfoliation and hydrazine-assisted ball milling. These parameters are arranged according to the dispersion force, δ_d_, polarity cohesion, δ_p_, and hydrogen bonding cohesion, δ_h_, of the dispersion [35]. Our NFs obtained through milling significantly increased the sum value of δ_p_ and δ_h_, in the range of 15–30.3, whereas MoS_2_ in a previous report had a range of 10.5–20.5, indicating that our NFs have improved dispersibility [36]. As shown in Figure S9, the values of all parameters were increased compared to the previous report [36]—in particular, the degree of increase in δ_p_ and δ_h_ is larger than the degree of increase in δ_d_. This finding suggests that polarity and hydrogen bonding are more dominant than the dispersion force for the oxygen functional group attached to NFs during milling, allowing the dispersibility of MoS_2_ NFs even in relatively highly polar solvents.

### 3.4. Photodetector Using MoS_2_ Nanoflakes

**Figure 4:**
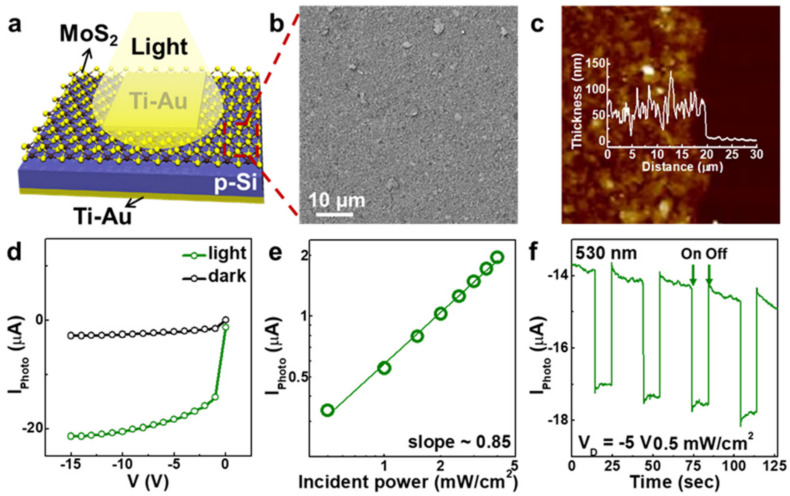
(**a**) Schematic illustration, (**b**) scanning electron microscopy images, and (**c**) atomic force microscopy (AFM) topography image of the MoS_2_–p-Si diode. Height profiles are shown in the AFM image. (**d**) Current–voltage (I–V) characteristics in the dark and under illumination. (**e**) Time-dependent on/off photocurrent measured at −5 V and 0.5 mW/cm^2^. (**f**) Photocurrent as a function of time.

To explore the possibility of applying this technology to an optoelectronic device, a vertical MoS_2_–p-Si stack device structure was designed and characterized. Figure 4a and Figure S10 present the schematic and current–voltage (I–V) characteristics of the MoS_2_–p-Si heterojunction diode, respectively. Au/Ti films (70/5 nm) were deposited for use as the top and bottom electrodes. A p–n junction was made using a Fermi-level alignment between p-type Si and n-type MoS_2_. Typical rectifying behavior was observed in the I–V measurements, with a significant current increase under forward bias, but a reduced current under reverse bias (Figure S10). Figure 4b,c present SEM and AFM images of the MoS_2_ layer placed on a p-Si substrate synthesized using the Langmuir–Blodgett method. The MoS_2_ NFs were uniformly coated on a p-Si substrate without cracks, and the thickness of the MoS_2_ layer was estimated to be ~70 nm.

Figure 4d compares the electrical characteristics of the MoS_2_–p-Si diode under dark and illuminated conditions. The current measurement was conducted while voltage was swept in the range of 0 to −15 V. The photodetector exhibited the typical photoconductive behavior of a conventional p–n junction diode. When exposed to visible light, the drain current changed from −2.8 to −21.4 μA at −15 V, indicating significant photocarrier generation. To further investigate the photo-sensing behavior of the diode, the photocurrent was measured under light with various intensities (0.5–4.0 mW/cm^2^; Figure 4b). The drain current increased as the light power did so, following the power-law relationship:

I_PC_ ∝ P^0.85^ (1)

where I_PC_ is the photocurrent and P is the power of the light. Our photodiode exhibits linear behavior in a log(I_PC_)–log(P) plot, which can be attributed to the photoelectric effect. The power-law exponent from our device is ~0.85, which is higher than that of other materials such as graphene or ZnO nanowires [37,38,39].

A time-dependent on/off photoconduction measurement of the diode was also performed to estimate its switching speed under light exposure. We applied −5 V of drain voltage to the device and light intensity was maintained at 0.5 mW/cm^2^. The measurement was conducted with 10 s light on and 20 s light off, and the sequence was repeated four times. As shown in Figure 4f, fast switching speed is observed with the green light on/off switching. The photodiode had a quick rise time, of 0.1 s, after turning on the light. Furthermore, a significant sharp decrease in current was observed, with a 0.15 s decay time, after the light was turned off. Photo-sensing measurements were also completed under other wavelengths (400 and 850 nm; Figure S11). The photodiode even responded to 850 nm, indicating that the MoS_2_ NFs obtained through our process have only a few layers, so the diode has the ability to detect low-energy light. Overall, the MoS_2_–p-Si photodiode exhibits a good response to visible and near-infrared radiation.

## 4. Conclusions

In summary, we present a method for easily fabricating MoS_2_ NFs using hydrazine-assisted ball milling via the synergetic effect of chemical intercalation and mechanical peeling. MoS_2_ NFs with 600−800 nm size and a thickness of a few layers were obtained with little damage to their structure. In the hydrazine-assisted ball milling process, an oxygen functional group is attached to NFs, and this functional group enables stable dispersibility in various solvents. The high stability in various solvents can be easily applied to solution-based processes and used in numerous applications. Furthermore, to demonstrate a photonic application of the high-quality 2H-MoS_2_, heterojunction photodiode devices were fabricated using the Langmuir–Blodgett coating process and evaluated. The MoS_2_ photodiode exhibited stable photo-sensing characteristics in radiation with wavelengths of 850, 530, and 400 nm, with excellent photo-switching characteristics at all wavelengths. Thus, our methods can be applied to exfoliation of other transition-metal dichalcogenides by using proper chemical intercalants, as well as high performance MoS2-based photodiodes having potential for use in practical photonic applications.

## Supplementary Materials

The following are available online at https://www.mdpi.com/2079-4991/10/6/1045/s1, Figure S1: TEM images for MoS_2_ NFs with varying rpm, Figure S2: TEM images for MoS_2_ NFs according to milling tine, Figure S3: Raman spectra of MoS_2_ NFs, Figure S4: XPS spectra of as-received MoS_2_, Figure S5: XPS spectra of MoS_2_ NFs, Figure S6: TGA curves of MoS_2_ NFs, Figure S7: X-ray diffraction pattern of as-received MoS_2_ and MoS_2_ NFs, Figure S8: Dispersion properties and optical bandgap of MoS_2_ NFs, Figure S9: Hansen solubility parameter of MoS2 NFs, Figure S10: I-V characteristic of MoS_2_/p-Si diode, Figure S11: Time-dependent on/off photo current with various wavelength.
