# Supplementary material for: Novel Exfoliation of High-Quality 2H-MoS2 Nanoflakes for Solution-Processed Photodetector
Source: Nanomaterials (Basel). 2020 May 29;10(6):1045. doi: 10.3390/nano10061045 (PMC7352925; doi:10.3390/nano10061045)
Supplement: Supplementary file 1 [file nanomaterials-10-01045-s001.pdf]

nanomaterials

Supplementary Materials

Novel Exfoliation of High-Quality 2H-MoS_2 Nanoflakes for Solution-Processed Photodetector

Seulgi Kim [1,†], Woojin Park [1,†], Dohoon Kim [1], Jiyeon Kang [1], Jaesoung Lee [1], Hye Yeon Jang [1], Sung Ho Song [2,*], Byungjin Cho [1,*] and Dongju Lee [1,*]

[1] Department of Advanced Materials Engineering, Chungbuk National University, Chungdae-ro 1, Seowon-Gu, Cheongju 28644, Korea; kimsg@chungbuk.ac.kr (S.K.); wjpark80@gmail.com (W.P.); rlaehgns235@gmail.com (D.K.); wldusk0609@chungbuk.ac.kr (J.K.); jaesunglee@cbnu.ac.kr (J.L.); hyjang0581@gmail.com (H.Y.J.)

[2] Division of Advanced Materials Engineering, Kongju National University, Kongju, Chungnam 330-717, Korea

* Correspondence: shsong805@kongju.ac.kr (S.H.S.); bycho@chungbuk.ac.kr (B.C.); dongjulee@chungbuk.ac.kr (D.L.)

† These authors contributed equally to this work.

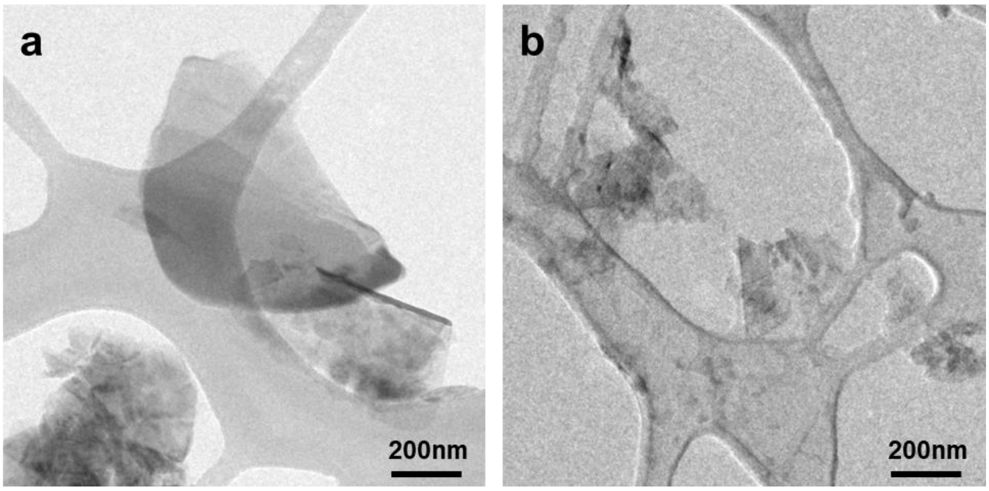

Figure S1. TEM images for MoS_2 NFs with varying rpm. (**a**) 100 rpm, (**b**) 300 rpm.

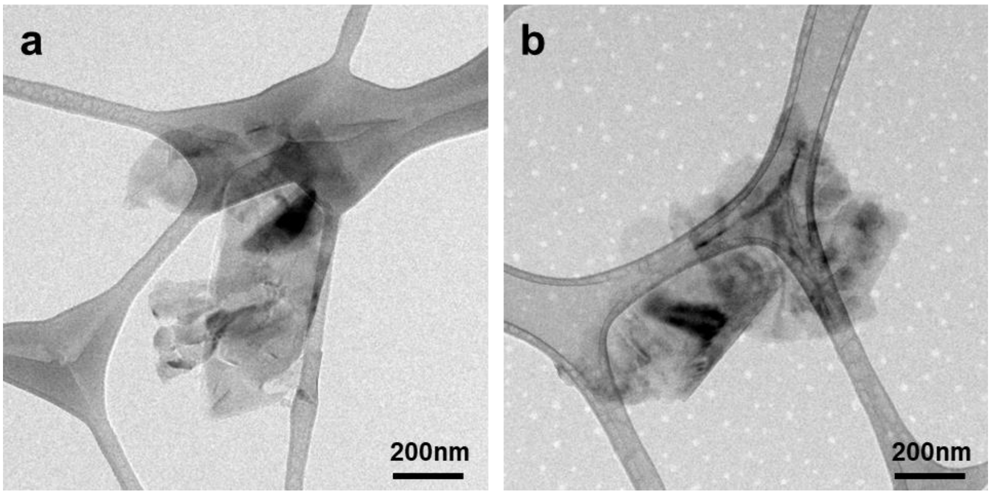

Figure S2. TEM images for MoS_2 NFs according to milling tine. (**a**) 24 hr, (**b**) 36 hr.

The Raman spectra of the MoS_2 NFs were examined using a 532 nm laser source. Figure S3 shows peaks corresponding to the typical A_{1g} mode and E^1_{2g} mode of MoS_2 at 407.05 cm^{-1} and 384.04 cm^{-1}, respectively. A_{1g} mode and E^1_{2g} mode cause a blue shift and red shift respectively when the number of MoS_2 layers decreases. Thus, the number of MoS_2 layers can be judged by intervals between the peaks of the two modes.

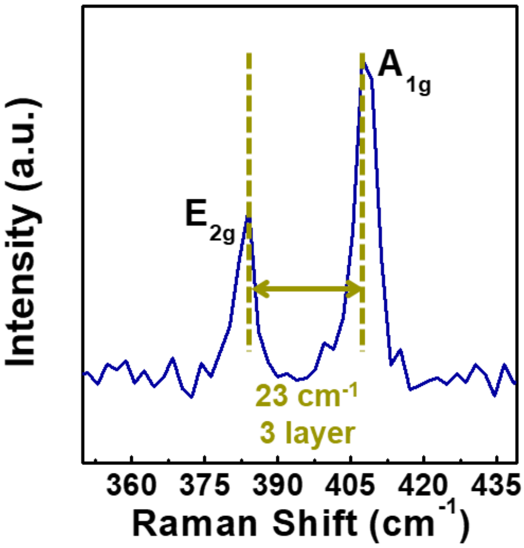

Figure S3. Raman spectra of MoS_2 NFs.

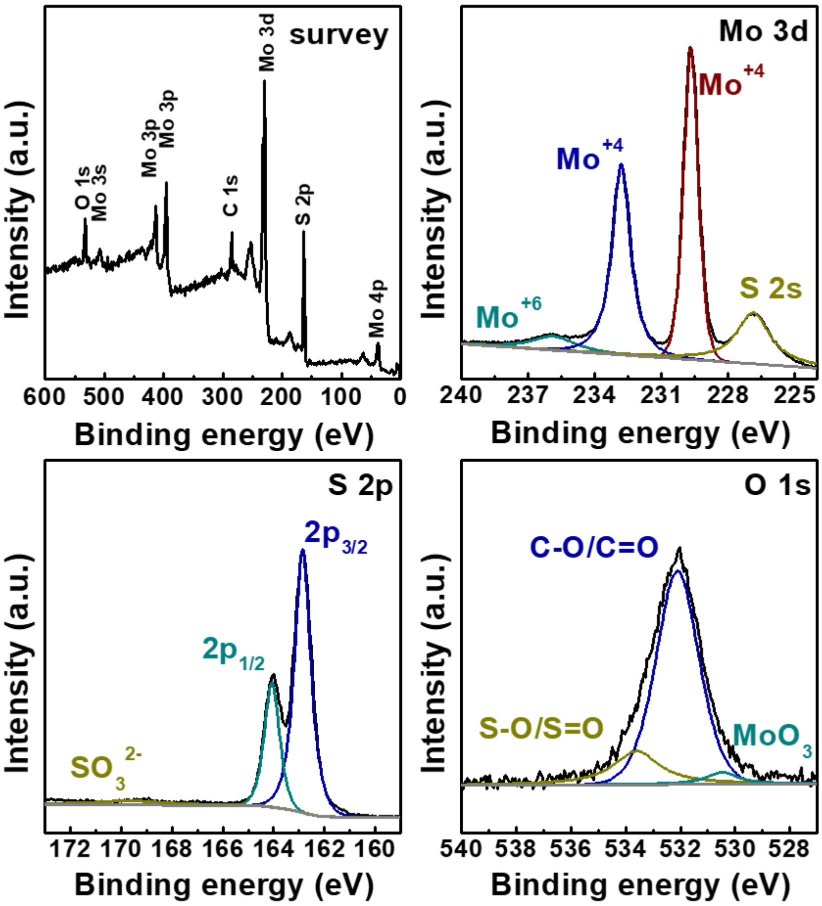

Figure S4. XPS spectra of as-received MoS_2 (**a**) survey scan, (**b**) Mo 3d, (**c**) S 2p and (**d**) O 1s narrow scan.

Fe residues to be formed during the ball milling process can be confirmed by the Fe narrow scan results in Figure S5b. It is likely that the Fe ion and contaminations perfectly get washed by HCl solution.

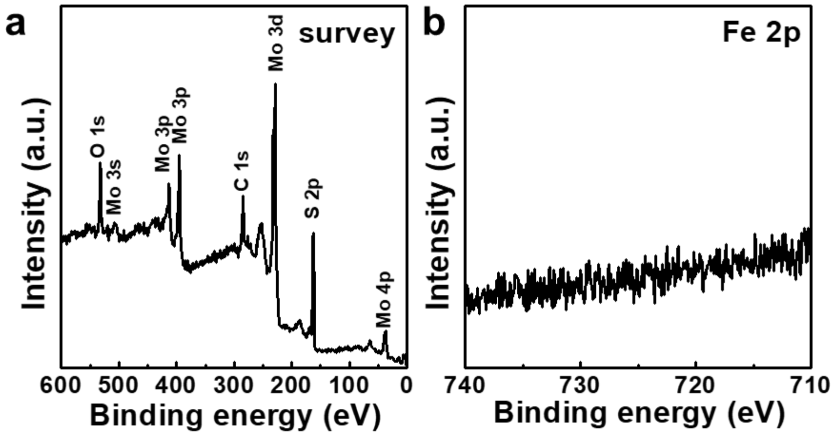

Figure S5. XPS spectra of MoS_2 NFs (**a**) XPS survey scan, (**b**) Fe narrow scan.

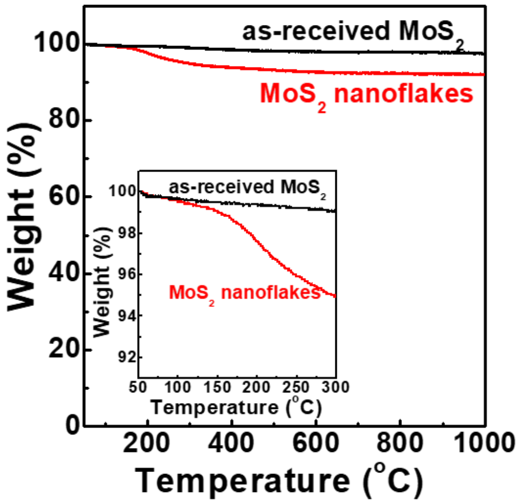

Figure S6. TGA curves at 10°C /min for the thermal decomposition in N_2 atmosphere of MoS_2 NFs.

X-ray diffractometer (XRD) was examined to further identify the crystal structure of MoS_2 NFs. As shown in figure S3, the XRD result show that the MoS_2 NFs have a 2H structure even after the hydrazine-assisted ball milling process. In addition, all peaks became smaller and broader than as-received MoS_2 peaks, indicating that the lateral size of MoS_2 is smaller after exfoliation process.

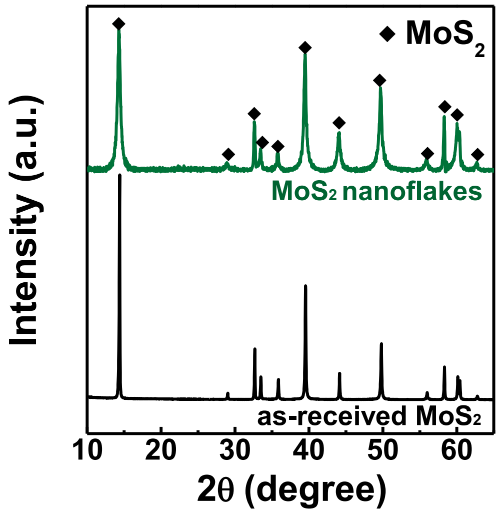

Figure S7. X-ray diffraction pattern of as-received MoS_2 and MoS_2 NFs.

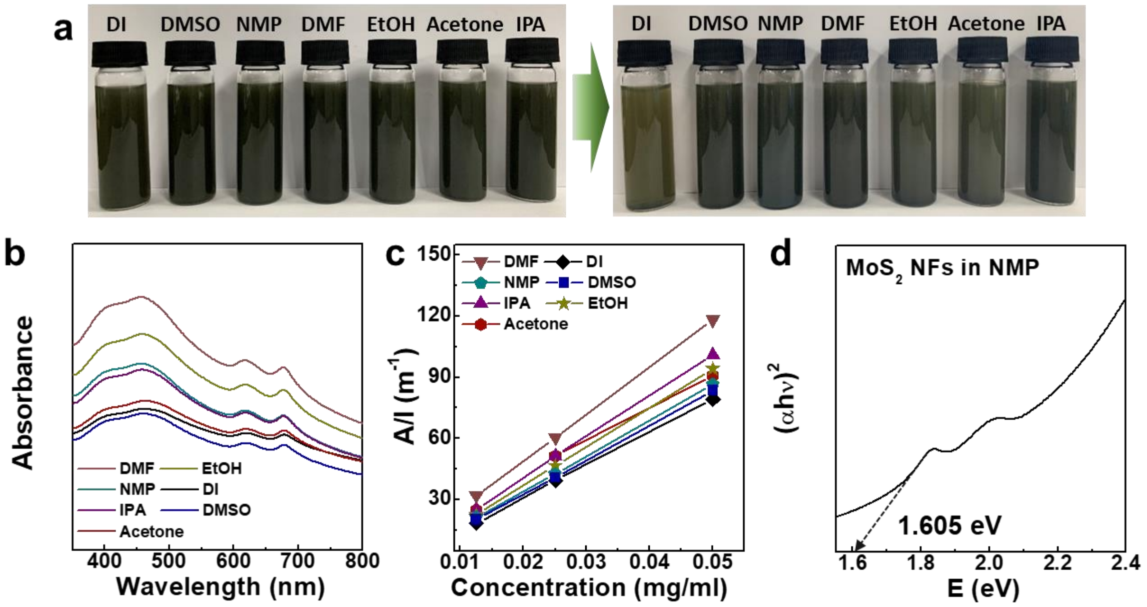

Figure S8. (**a**) Digital images of MoS_2 NFs suspension in various solvents for 1 week. (**b**) UV-vis absorption spectrum for MoS_2 NFs. (**c**) Optical absorbance slopes at excitation wavelength 670 nm as a function of the MoS2 NFs concentration in each solvent showing Lambert–Beer behavior. (**d**) Tauc plot of MoS_2 NFs.

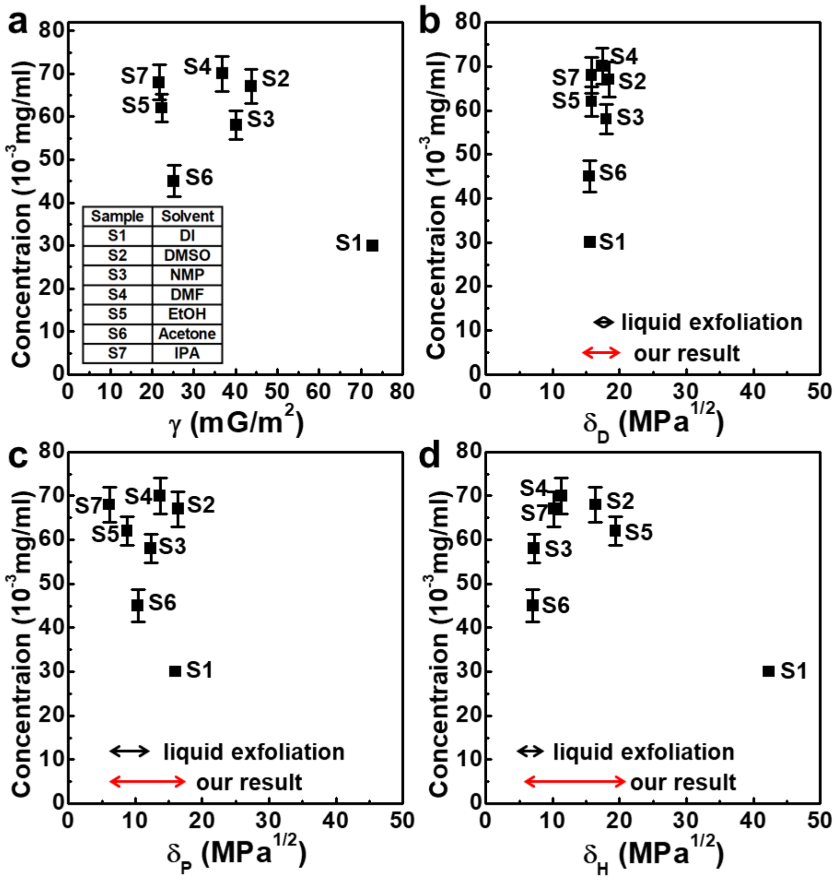

Figure S9. (**a**) Dispersibility of MoS_2 NFs in various solvents and their surface tension. Dispersion concentration of MoS_2 NFs plotted as a function of the (**b**) dispersive, (**c**) polarity, and (**d**) hydrogen bonding.

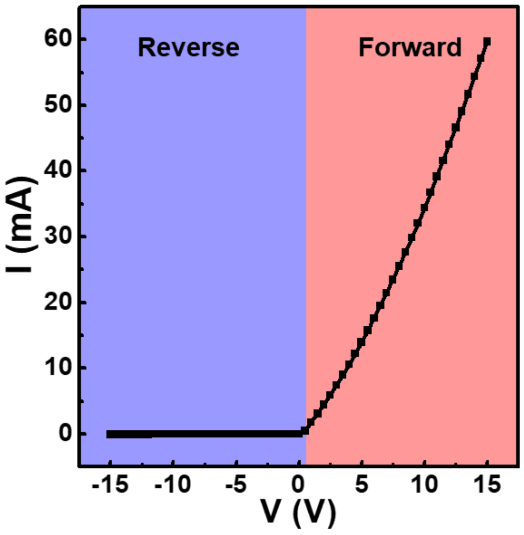

Figure S10. I-V characteristic of MoS_2/p-Si diode.

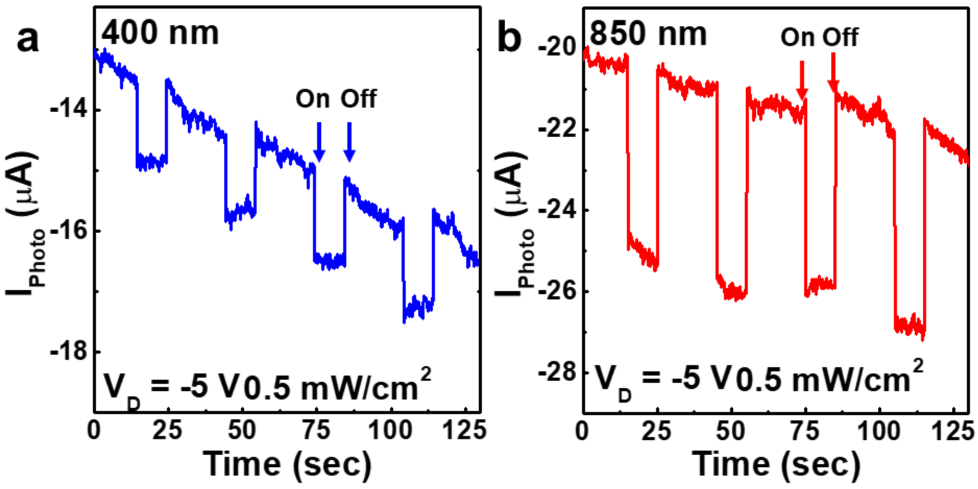

Figure S11. Time-dependent on/off photo current with (**a**) 400 nm and (**b**) 850 nm wavelength.
